# Combined methods reveal task activation dynamics in human brain networks

**DOI:** 10.1371/journal.pbio.3001749

**Published:** 2022-08-19

**Authors:** Zach Ladwig, Yuhua Yu, Caterina Gratton

**Affiliations:** Department of Psychology, Northwestern University, Evanston, Illinois, United States of America

## Abstract

This Primer explores a new study in PLOS Biology that combines the millisecond temporal resolution of EEG with MRI-based source localization and multivariate modeling to track dynamic task representations in human brain networks throughout a trial.

## Teaser

A clear understanding of how human brain networks reflect task performance has been lacking, in part due to methodological difficulties. A new study combines the temporal resolution of EEG, MRI source localization, and multivariate modeling to address this need.

Complex cognitive functions are carried out by the activity of billions of neurons organized into large-scale networks in the human brain. It is a primary aim of modern neuroscience to understand how cognitive information is represented in this system. In recent years, researchers have made some progress towards this goal. Using functional magnetic resonance imaging (fMRI), researchers have been able to track task information distributed across widespread brain regions [1,2]. In a recent paper in *PLOS Biology*, Mill and colleagues [3] expand on this work by merging modalities and models, providing a dynamic picture of how task representations in brain networks evolve across a trial.

A comprehensive picture of task representations and how they relate to human brain networks over time has historically been difficult due to key limitations in noninvasive imaging methods. fMRI provides relatively good spatial resolution and coverage, allowing for the delineation of networks at millimeter scale across the whole brain, but it is too slow to capture high-frequency activity. Electroencephalography (EEG) is fast, measuring electrical neural signals at the rate of milliseconds, but it is difficult to localize EEG signals to specific brain regions. Because animal literature from invasive intracranial recordings suggests that task information is encoded at fine temporal and spatial scales [4], advances in pushing the spatiotemporal resolution of human research are critically important. This more detailed spatiotemporal pattern can then serve as the basis to model how brain activity encodes task information, providing insight into underlying mechanisms.

**Fig 1:**
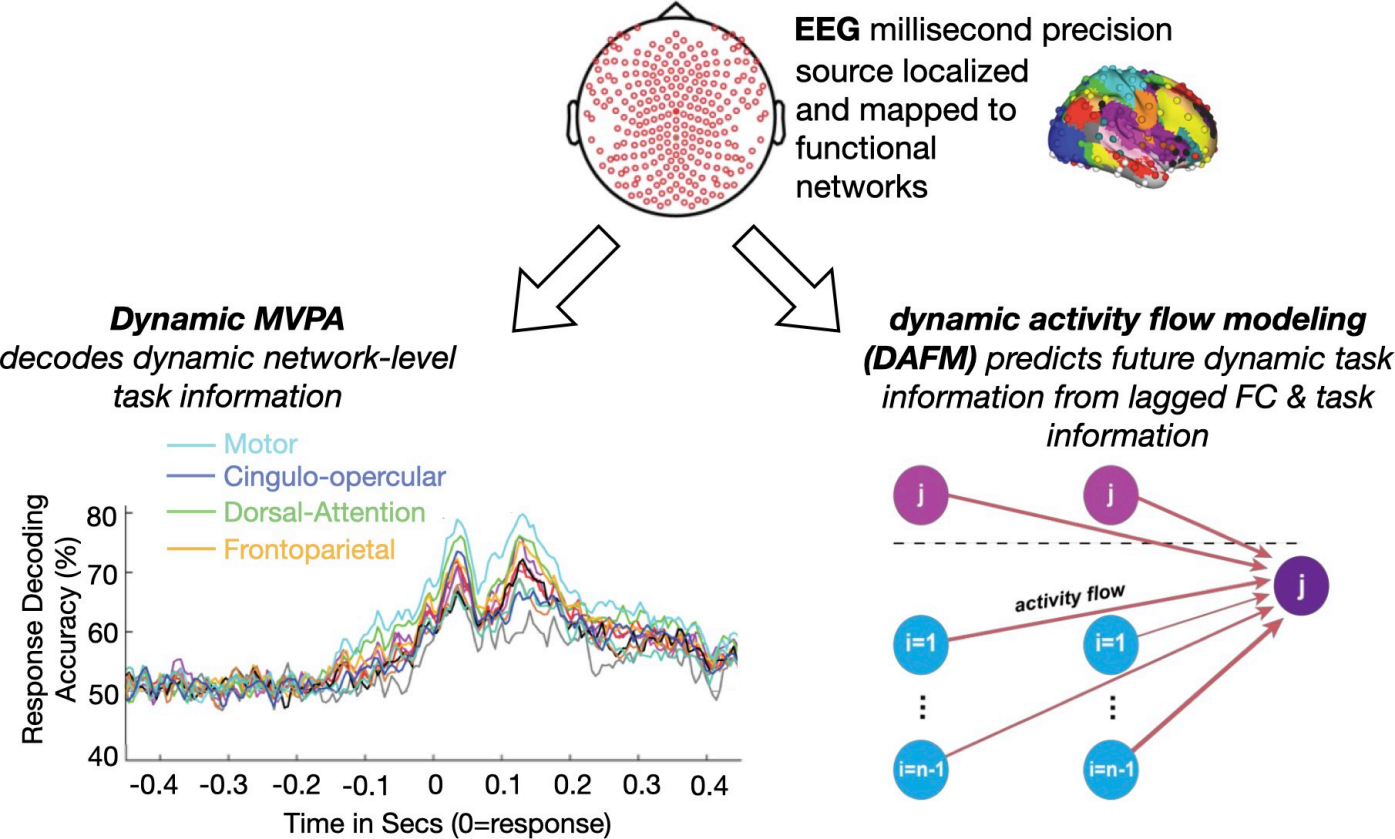
Mill and colleagues [3] study brain network interactions in task representations by combining EEG task and rest information source modeled to functional networks from previous fMRI results. Using MVPA in a sensory-motor task, they decode task information over time per network, finding motor and cognitive control networks are particularly prominent in decoding accuracy and temporal onset. Using DAFM, they predict future EEG task activity from lagged resting state EEG patterns and task activity. DAFM, dynamic activity flow modeling; EEG, electroencephalography; MVPA, multivariate pattern analysis.

In their new study, Mill and colleagues [3] present a modeling approach (Fig 1) to investigate spatiotemporal task representations using EEG connectivity estimates, which were source localized and mapped onto networks previously defined with fMRI. This method represents an important advance in our ability to characterize where, when, and how brain network activity is related to task states.

Previous work by Cole and colleagues [5] used brain network structure from resting state fMRI—shown to be largely stable within people across tasks [6]—to create an activity flow model in which task-evoked fMRI activity from one region was predicted from contemporaneous activity in another. This work was successful in predicting task activations, but due to the slow nature of fMRI, it could not make strong claims about the directional pattern of activity spread. In the current work, Mill and colleagues [3] extend their model to EEG with dynamic activity flow modeling (DAFM). DAFM provides a directed connectivity structure from the resting state (in this case, EEG signal) by estimating the activity in a target region using time-lagged signals from other regions. This pathway can be used to predict the activity in a response-generating (e.g., motor) region when loaded with prior task-evoked activity from other regions.

To study how brain networks support task representations, the authors brought in multivariate pattern analysis (MVPA). These multivariate techniques have been fruitfully applied to fMRI data to reliably discriminate task information based on brain activity patterns (e.g., visual versus auditory stimuli, or left versus right hand response) [1]. However, even the simplest cognitive task involves multiple steps, where the task representation unfolds in the brain across distributed networks. The current paper highlights the temporal dimension of task representations in each brain network by applying MVPA to EEG data that were source localized and projected onto brain networks previously defined via fMRI.

Source localization was key to overcoming the limitation of spatial ambiguity in EEG data in this work. The authors used anatomical MRI data to obtain individualized head models that allow for neural source localization with fine granularity [7]. The sources were then associated with a whole-brain map of 264 regions organized into 11 well-validated large-scale brain networks [8]. Mill and colleagues [3] were able to use this data to reveal that, while task representation can be recovered in all major functional networks, they emerge in a graded fashion, both in terms of their temporal order and decoding accuracy (**Fig 1**). This result balances the view of network specialization and spatially distributed task involvement.

Combining multivariate pattern classification and DAFM, the authors provide a powerful method to infer how task information propagates across the brain. Using the pathway estimated from resting-state EEG data, task-evoked activity in nonmotor regions not only predicts motor region responses but also can be used to decode response choice. The authors further conducted a lesion simulation, which selectively removed (“lesioned”) all but one functional network. By comparing the resulting task representation accuracy and how it evolved dynamically at a sub-second timescale, the authors inferred the relative contributions of each network towards response-relevant computation. This approach opens a window to understanding the impact of any individual region on the whole brain network.

Future studies will be critical to expand on the potential of this work. First, while the spatial resolution here is significantly better than most previous EEG studies, it is still lower (network level) and dependent on assumptions (source modeling) relative to invasive electrophysiological recordings and fMRI. Future improvements to DAFM may come from grounding the model in robust high-spatial resolution data such as human electrocorticography and through continued improvements in source modeling techniques. Second, the authors focused on decoding a relatively simple sensory-motor task in this proof-of-concept article. Future applications will be needed to explore how this model delineates spatiotemporal network activity in more complex task contexts. Finally, the simulated lesions imposed by the authors suggest that an exciting avenue of future work will be to pair this model with recordings from patients with brain damage or participants undergoing brain stimulation.

In sum, Mill and colleagues [3] demonstrate a significant step forward in overcoming spatiotemporal limitations of noninvasive human imaging by merging modalities and methods. Through this approach, the authors are able to present a dynamic view of brain network activity during tasks. This novel approach provides a roadmap for how to jointly answer where, when, and how questions regarding human brain function.

## Abbreviations:

DAFM: dynamic activity flow modeling
EEG: electroencephalography
fMRI: functional magnetic resonance imaging
MVPA: multivariate pattern analysis
